# Social Disadvantage and Multimorbidity Including Oral Conditions in the United States

**DOI:** 10.1177/00220345241228834

**Published:** 2024-03-19

**Authors:** A. Mirza, R.G. Watt, A. Heilmann, M. Stennett, A. Singh

**Affiliations:** 1 Department of Epidemiology and Public Health, University College London, London, UK; 2 Centre for Epidemiology and Biostatistics, Melbourne School of Population and Global Health, University of Melbourne, Melbourne, Victoria, Australia; 3 Melbourne Dental School, University of Melbourne, Melbourne, Victoria, Australia

**Keywords:** chronic diseases, oral health, health inequalities, socioeconomic factors, cross-sectional studies, NHANES

## Abstract

Existing studies on multimorbidity have largely excluded oral diseases in multimorbidity prevalence estimates. The reason behind this is somewhat unclear, as chronic oral conditions are highly prevalent, affecting over half the global population. To address this gap, we examined the relationship between social disadvantage and multimorbidity, stratifying by the inclusion and exclusion of oral conditions. For participants aged 30 y and over (*n* = 3,693), cross-sectional analysis was carried out using the US National Health and Nutrition Survey (2013–2014). Multimorbidity was defined as having 2 or more chronic conditions. Five medical conditions were examined: diabetes, asthma, arthritis, cardiovascular disease, and depression, as well as 4 oral health conditions: caries, periodontal disease, number of teeth, and edentulousness. Education and income poverty ratio were selected as measures of social disadvantage. Multimorbidity prevalence estimates according to social disadvantage were analyzed on an absolute and relative scale using inverse probability treatment weighting (IPTW), adjusting for age, sex, and ethnicity. The inclusion of oral health conditions in the assessment of multimorbidity increased the overall prevalence of multimorbidity from 20.8% to 53.4%. Findings from IPTW analysis demonstrated clear social gradients for multimorbidity estimates stratified by the exclusion of oral conditions. Upon inclusion of oral conditions, the prevalence of multimorbidity was higher across all social groups for both education and income. Stratifying by the inclusion of oral conditions, the mean probability of multimorbidity was 27% (95% confidence interval [CI], 23%–30%) higher in the low-education group compared to the high-education group. Similarly, the mean probability of multimorbidity was 44% (95% CI, 40%–48%) higher in the low-income group. On a relative scale, low education was associated with a 1.52 times (95% CI, 1.44–1.61) higher prevalence of multimorbidity compared to high education. Low income was associated with a 2.18 (95% CI, 1.99–2.39) higher prevalence of multimorbidity. This novel study strongly supports the impact of chronic oral conditions on multimorbidity prevalence estimates.

## Introduction

Multimorbidity is a growing public health issue impacting millions of individuals worldwide (Academy of Medical Sciences 2018). Whilst a universal definition does not exist, multimorbidity is commonly defined as the coexistence of 2 or more chronic conditions (WHO 2016; National Institute of Clinical Excellence 2023). In the United States, it is estimated that 43% of the population have multimorbid conditions (Chowdhury et al. 2023). In low and middle-income countries, the prevalence of multimorbidity continues to rise, affecting nearly a fifth of the population (Berner et al. 2022). The impact of multimorbidity can be seen at an individual and broader socioeconomic level, determining quality of life, health service utilization, and economic productivity (Barnett et al. 2012). Whilst multimorbidity is widely associated with an aging population, social disadvantage is also an important determinant of multimorbid conditions (Fleitas Alfonzo et al. 2022). As multimorbidity gains increased political and social interest, the need for high-quality research is becoming increasingly important. Currently, our understanding of multimorbidity remains limited, hampered by the absence of a universal definition and major gaps in research (Academy of Medical Sciences 2018).

Chronic oral diseases affect 3.5 billion people worldwide, causing physical and social impairments such as pain, eating difficulties, and low self-esteem (WHO 2022). The enormous challenges presented by oral diseases are clearly demonstrated by the findings of the Global Burden of Disease Study (2019), which reported untreated tooth decay in the permanent dentition as the most prevalent chronic condition worldwide, affecting an estimated 29% of the global population (Institute for Health Metrics and Evaluation 2020). Furthermore, the prevalence of severe periodontitis, a major cause of tooth loss, also continues to rise, impacting almost 20% of the global population (WHO 2022). Despite the global prevalence of oral diseases, they are seldom included in the broader category of chronic health conditions. This is perhaps surprising, as modifiable risk factors such as diet, smoking, and alcohol and their underlying social and commercial determinants are common to both oral and general health (Watt and Sheiham 2012). Nevertheless, oral conditions remain typically isolated from the broader, generic definition of chronic disease and are rarely considered from a multimorbidity perspective (Chua et al. 2021).

Studies on multimorbidity have primarily focused on medically diagnosed chronic conditions. A recent scoping review on the prevalence of multimorbidity found highly variable estimates, ranging from 15.3% to 89.7% (Chua et al. 2021). Of the 20 studies reported on and 21 health systems examined, the scoping review failed to consider any oral conditions in prevalence estimates of multimorbidity. Similarly, a recent systematic review on multimorbidity and socioeconomic status analyzed 5 to 334 chronic health conditions (Pathirana and Jackson 2018), but from the data provided, oral conditions were not reported on. A few studies on multimorbidity and chronic dental diseases have examined oral health outcomes such as tooth loss (Bomfim et al. 2021; Casanova-Rosado et al. 2021), caries, and periodontal disease (Islas-Granillo et al. 2019). We identified only 1 study that reported periodontal disease within multimorbidity prevalence estimates, but no detailed analysis was carried out inclusive of this estimate (O’Dwyer et al. 2023).

Socioeconomic inequalities are well documented, with groups experiencing social disadvantage at higher risk of worse health outcomes. A systematic review examining socioeconomic status and multimorbidity (Pathirana and Jackson 2018) found that those with low education compared to those with high education were 64% more likely to be multimorbid. Living in an area of high deprivation was also significantly associated with a higher risk of multimorbidity. The relationship between multimorbidity and income remains somewhat unclear. Studies conducted in high-income countries have reported conflicting results. In the United States, an association between income and multimorbidity was found in unadjusted analyses only (Tucker-Seeley et al. 2011). However, in a Canadian study, a positive relationship was found (Agborsangaya et al. 2012). In contrast, there is some evidence to suggest high income is significantly associated with an increased risk of multimorbidity in low-income countries (Alaba and Chola 2013).

To address this current research gap, the aim of this study was to examine the relationship between social disadvantage and multimorbidity, stratifying by the inclusion and exclusion of oral conditions. Due the prevalence of oral conditions and steep social gradients (WHO 2022), we hypothesized that the inclusion of oral conditions within multimorbidity estimates would increase multimorbidity prevalence and the magnitude of inequalities.

## Materials and Methods

This study is reported in accordance with STROBE guidelines (Appendix Fig. 1).

### Data and Analytical Sample

We analyzed cross-sectional data from the nationally representative US 2013 to 2014 National Health and Nutrition Survey (NHANES) (Centers for Disease Control and Prevention 2023). NHANES is a complex, multistage probability survey of non-institutionalized US civilians, conducted by the National Center for Health Statistics. Data are self-reported and recorded electronically via Computer Assisted Personal Interviewing (CAPI) and Audio Computer-Assisted Self-Interview (ACASI). Participants also undertake a standardized physical examination at a mobile examination center. Further details regarding NHANES data, sampling protocols, and technical information can be obtained at https://www.cdc.gov. Ethical approval for 2013 to 2014 NHANES was granted by the National Center for Health Statistics Research Ethics Review Board.

The 2013 to 2014 NHANES data were analyzed due to the availability of both oral health and medical data. The analytical sample was restricted to participants aged 30 y and over; please see participant flow diagram Appendix Figure 2. Statistical analyses were based on complete case analysis, resulting in a final sample size of 3,693 participants.

### Outcome

Multimorbidity was defined as the presence of 2 or more chronic health conditions (WHO 2016). Two multimorbidity outcomes were considered: multimorbidity assessment excluding oral diseases (only medical conditions) and multimorbidity assessment including oral diseases (medical and dental conditions). Multimorbidity was analyzed as a binary variable coded 1 if multimorbidity was present and 0 if multimorbidity was absent. For the assessment of multimorbidity, 5 medical conditions and 4 oral health conditions were considered. Similar to US data (Goodman et al. 2016; Jindai et al. 2016), we considered diabetes, asthma, arthritis, cardiovascular disease (CVD), and depression. Oral health measures were dental caries, periodontal disease, number of teeth, and edentulousness.

In NHANES (2013–2014), data on chronic medical conditions are self-reported via the following question: “Has a doctor or other health professional ever told you that you have. . . ?” Response categories are “yes,” “no,” “don’t know,” and “refused.” We created binary variables for diabetes, asthma, and arthritis, coded 1 = disease present and 0 = disease absent. Similar to Moonesinghe et al. (2019), we also created a binary CVD variable, 1 = CVD and 0 = no CVD, using the available medical data. Participants who responded “yes” to any of the following conditions were included in the CVD variable: stroke, angina, congenital heart disease, and heart failure. To assess symptoms of depression, NHANES uses the 9-item Patient Health Questionnaire (PHQ-9) (Kroenke et al. 2001) (Appendix Fig. 3). Each of the 9 questions ise given a score of 0 to 3 based on the following responses: “not at all,” “several days,” “more than half the days,” and “nearly every day.” Responses were summed to obtain a PHQ-9 score ranging from 0 to 27 and dichotomized as no/mild depression (score 0–9) versus moderate/severe depression (score 10 or more) using agreed cutoff criteria (Manea et al. 2012).

Dental examinations were conducted at the mobile examination center, equipped with a dental chair, artificial halogen light, and compressed air for tooth drying. Between NHANES 2011 and 2014, oral health data were primarily collected by 3 licensed, calibrated dentists, using the NHANES protocol for oral health examinations (Dye et al. 2019). Complete protocol information can be accessed via the NHANES 2013 Oral Health Examiners Manual: https://wwwn.cdc.gov/nchs/data/nhanes/2013-2014/manuals/Oral_Health_Examiners.pdf. We defined caries as untreated coronal decay in the permanent dentition and coded caries 1 = yes and 0 = no. Number of teeth was categorized as fewer than 20 teeth (nonfunctional dentition) versus 20 or more teeth (functional dentition) based on World Health Organization (WHO) guidance (WHO Expert Committee on Recent Advances in Oral Health & World Health Organization 1992). Complete tooth loss was categorized as edentulousness (no teeth) versus nonedentulousness (1 or more natural teeth present). Third molars were excluded from analysis.

A full-mouth periodontal examination was carried out for participants aged 30 or over who had at least 1 tooth present. Periodontal data were collected on gingival recession and pocket depth using 6 points at the tooth surface (distal-buccal, mid-buccal, mesio-buccal, distal-lingual, mid-lingual, and mesio-lingual). Based on guidance provided by the Centers for Disease Control and Prevention and American Academy of Periodontics (Eke et al. 2012), periodontal disease was defined as having a minimum of either 2 or more interproximal sites with loss of attachment of ≥3 mm and 2 or more interproximal sites with probing depths of ≥4 mm (not on the same tooth) or 1 or more interproximal sites with probing depths of ≥5 mm. We categorized periodontal disease as 1 = periodontal disease present and 0 = periodontal disease absent. For caries, periodontal disease, and number of teeth, a separate category was created for those who were edentulous, to retain them in the analysis.

### Exposure

We selected 2 measures of social disadvantage, education and income poverty ratio. Similar to Aldosari et al. (2020), education was categorized as low education (less than high school), medium education (high school/General Education Development high school equivalency test), and high education (more than high school). Based on previous NHANES data, income poverty ratio was also categorized as 3 groups: low (<100%), medium (>100%–400%), and high (>400%) (Odutayo et al. 2017).

### Covariates

All covariate data were self-reported and collected by CAPI. Based on the existing literature, the following covariates were considered for analyses: sex (male or female), age (30–39, 40–54, 55–65, and 65+ y), marital status (married/living with partner or single), and race/ethnicity (non-Hispanic White, Mexican American/other Hispanic, non-Hispanic Black, and other). Smoking status was categorized as nonsmoker, current smoker, and former smoker.

### Statistical Analyses

All statistical analyses were performed using STATA version 17.1 from StataCorp LP. NHANES 2-y Mobile Examination Center (MEC) examination weights and cluster/strata sampling design variables were applied to the data to account for complex survey design. Complete case analysis was conducted (*n* = 3,693), excluding those with missing data on variables of interest. Descriptive analyses were carried out to determine sample characteristics. Descriptive characteristics were also analyzed for those with and without missing data, to identify any significant differences between the 2 population groups. Inverse probability treatment weighting (IPTW) was used to examine the relationship between social disadvantage and multimorbidity. We used IPTW to account for confounding factors (age, sex, and ethnicity) and create a pseudo population, maximizing exchangeability between the exposed group (those experiencing social disadvantage) and unexposed group (those not experiencing social disadvantage). In comparison to traditional regression models, by applying IPTW, the prevalence of multimorbidity according to social disadvantage was assessed on both an absolute and a relative scale, a recommended practice for reporting social inequalities in health outcomes (King et al. 2012). Absolute measures of multimorbidity indicate the overall magnitude of inequality between social groups, whereas relative measures indicate proportional health differences across social groups (Schlotheuber and Hosseinpoor 2022). We also obtained E-values for relative risk (RR) estimates, using the equation: RR + $\sqrt{RR}\times (RR-1)$ . E-values provide a quantifiable measure of residual confounding, a key source of study bias (Vander Weele and Ding 2017).

## Results

Table 1 shows descriptive characteristics of the study sample (*n* = 3,693) by multimorbidity. Mean participant age was 52.9 y (SD = 0.34), with an equal distribution of men and women. Those with high education and medium income formed the majority of the sample population. As would be expected, the prevalence of multimorbidity increased across age groups. The results show a clear social gradient, with higher prevalence of multimorbidity among lower socioeconomic groups. Those who were single and smoked were also more likely to be multimorbid. Participants excluded from analysis were more likely to be socially disadvantaged, smokers, and female (Appendix Table 1).

**Table 1.** Descriptive Characteristics of NHANES (2013–2014) by Multimorbidity *n*/Weighted Percentage (*n* = 3,693).

Characteristic	*n*	Multimorbidity Excluding Oral Conditions (*n* = 848, 20.8%), *n* (%)	Including Oral Conditions (*n* = 2,245, 53.4%), *n* (%)
Income**			
High	754	104 (12.7)	275 (33.1)
Medium	2,212	527 (23.1)	1,389 (57.8)
Low income	727	217 (29.1)	581 (79.0)
Education**			
High	2,142	426 (18.4)	1,070 (44.0)
Medium	814	195 (23.1)	587 (67.1)
Low	737	227 (29.1)	588 (77.4)
Sex^a^			
Male	1,790	339 (16.5)	1,092 (53.0)
Female	1,903	509 (25.0)	1,153 (54.0)
Age**			
30-39	773	61 (8.2)	323 (36.7)
40-49	771	92 (11.0)	354 (42.1)
50-64	1,147	319 (25.1)	762 (57.6)
65+	1,002	376 (35.8)	806 (73.6)
Ethnicity^b^			
White	1,728	439 (21.8)	1,028 (51.4)
Mexican	749	159 (15.4)	470 (55.0)
Black	745	186 (22.4)	535 (68.4)
Other	471	64 (17.8)	212 (48.7)
Marital status**			
Single	1,362	393 (28.0)	978 (65.8)
Married	2,331	455 (17.4)	1,267 (47.6)
Smoking status**			
No	1,968	383 (17.1)	1,016 (44.0)
Yes	777	203 (26.1)	594 (72.2)
Ex-smoker	948	262 (24.7)	635 (58.7)

a *P* < 0.05 for sex and multimorbidity excluding oral conditions; *P* > 0.05 sex and multimorbidity including oral conditions.

b *P* < 0.05 for ethnicity and multimorbidity excluding oral conditions; *P* < 0.001 for ethnicity and multimorbidity including oral conditions.

** *P* < 0.001.

The inclusion of chronic oral diseases in the assessment of multimorbidity considerably increased multimorbidity prevalence from 20.8% to 53.4%. An increase in the prevalence of multimorbidity was observed across all socioeconomic groups for both education and income levels. Table 2 shows the results of the IPTW analysis.

**Table 2.** Average Treatment Effects and Prevalence Ratios for the Relationship between Social Disadvantage and Multimorbidity.

Social Disadvantage^a^	Absolute Scale (ATE)	95% CI	Relative Scale (RR)	95% CI	E-value
Multimorbidity (chronic conditions without oral health conditions)					
Education					
High	Reference		Reference		
Medium	0.03	0.01–0.06	1.16	1.01–1.35	1.59
Low	0.08	0.05–0.12	1.39	1.22–1.60	2.13
Income					
High	Reference		Reference		
Medium	0.08	0.05–0.12	1.60	1.33–1.93	2.58
Low	0.16	0.12–0.21	2.15	1.76–2.64	3.72
Multimorbidity (chronic conditions with oral health conditions)					
Education					
High	Reference		Reference		
Medium	0.20	0.17–0.24	1.44	1.33–1.49	2.24
Low	0.27	0.23–0.30	1.52	1.44–1.61	2.41
Income					
High	Reference		Reference		
Medium	0.25	0.21–0.28	1.66	1.51–1.82	2.71
Low	0.44	0.40–0.48	2.18	1.99–2.39	3.78

ATE, average treatment effect; CI, confidence interval; RR, relative risk.

a Model adjusted for age, sex, and ethnicity.

Multimorbidity excluding oral diseases: After adjusting for confounding factors, clear associations between social disadvantage and multimorbidity were seen, according to education and income levels. Multimorbidity was 8% (95% confidence interval [CI], 5%–12%) higher in the low-education group compared to the high-education group. Therefore, if the whole population became socially disadvantaged, the probability of multimorbidity would increase by 0.08 units. Similarly, the mean probability of multimorbidity was 16% (95% CI, 12%–21%) higher in the low-income group compared to the reference group. On the relative scale, low education was associated with a 1.39 times (95% CI, 1.22–1.60) higher prevalence of multimorbidity, compared to high education. Low income was also associated with a 2.15 (95% CI, 1.76–2.64) higher prevalence of multimorbidity, compared to those who were socially advantaged.

Multimorbidity including oral diseases: For both education and income, the prevalence of multimorbidity appeared to be higher across all social groups with social gradients remaining constant. Mean probability of multimorbidity was 27% (95% CI, 23%–30%) higher in the low-education group compared to the high-education group. Similarly, the mean probability of multimorbidity was 44% (95% CI, 40%–48%) higher in the low-income group compared to the high-income group. On a relative scale, low education was associated with a 1.52 times (95% CI, 1.44–1.61) higher prevalence of multimorbidity. Low income was associated with a 2.18 (95% CI, 1.99–2.39) higher prevalence of multimorbidity, compared to high social advantage.

For both multimorbid groups (excluding and including oral health conditions), the results of bias analysis suggest plausibility of effect sizes on a relative risk scale. After adjusting for measured confounding factors, for low income and low education, we found E-value estimates greater than 2. This indicates the minimum magnitude of association between the confounding factor and exposure and between the confounding factor and outcome required to explain away the relationship between social disadvantage and multimorbidity. Therefore, the relative risk E-values substantiate the robustness of our study findings.

## Discussion

Nearly half the global population is affected by poor oral health, yet chronic oral conditions are seldom reported on in multimorbidity studies (Chua et al. 2021). Consequently, the results of this study are new and important, highlighting the importance of oral conditions on multimorbidity estimates. Unlike previous studies on multimorbidity, we considered 4 oral health conditions in multimorbidity prevalence estimates. Consequently, the overall prevalence of multimorbidity more than doubled. In light of these results, it may be argued that current multimorbidity prevalence estimates are underestimated when oral conditions are ignored.

Comparison of multimorbidity prevalence estimates is problematic due to a number of factors, including population age, sample size, and the number of chronic conditions reported on (Pathirana and Jackson 2018; Johnston et al. 2019). We identified a recent study by O’Dwyer et al. (2023) examining the relationship between multimorbidity and periodontal disease using NHANES data (2011–2014). In comparison, findings from this present study show a much lower prevalence of multimorbidity among the population sample (20.8% vs. 54.1%). Differences in prevalence estimates may be attributable to a number of factors, including the size of the study population (6,940 vs. 3,693) and the number of chronic conditions reported on. Inclusion of periodontal disease increased multimorbidity prevalence estimates from 54.1% to 65.8% (O’Dwyer et al. 2023). In this study, we reported a greater increase in the prevalence of multimorbidity upon inclusion of oral conditions, 20.8% to 53.4%. However, in comparison to O’ Dwyer et al. (2023), we reported a lower prevalence of multimorbidity, excluding oral health conditions, and also included 4 oral health conditions in multimorbidity estimates.

Unlike previous studies on socioeconomic status and multimorbidity (Pathirana and Jackson 2018), we considered multimorbidity estimates stratifying on exclusion and inclusion of oral conditions. Consequently, the findings of this study provide further evidence on social inequalities in both general and oral health, with disadvantaged groups at higher risk of multimorbidity. Study findings indicate social inequalities on both an absolute and a relative scale across both multimorbidity groups. However, the inclusion of oral conditions within the assessment of multimorbidity resulted in a clear difference in relative and absolute magnitude change. On a relative scale, upon inclusion of oral conditions, moderate increases in multimorbidity prevalence estimates were observed across all social groups for both education and income. However, on an absolute scale, a greater magnitude of inequality was observed for both education (0.08 vs. 0.27) and income (0.16 vs. 0.44). Absolute inequality measures are dependent on the prevalence and associated burden of disease; therefore, the overall prevalence of multimorbidity within the sample is an important driver of absolute estimates (UK Office for Health Improvement and Disparities 2023). Consequently, differences in absolute measures may be attributable to the higher prevalence of multimorbidity, including oral conditions (53.4% vs. 20.3%).

Due to our novel approach to social inequalities and the inclusion of oral diseases within multimorbidity assessments, a full, direct comparison of our results with previous studies is not possible. Nevertheless, the findings of this study can be used to support previous data on social disadvantage and multimorbidity, excluding oral health conditions. In line with the systematic review (Pathirana and Jackson 2018), we also found that those with low education, compared to high education, were markedly more likely to be multimorbid. Our study also supports findings from other high-income countries (Tucker-Seeley et al. 2011; Agborsangaya et al. 2012) on associations between income and health.

A major strength of this study is the presentation of new knowledge and furthering our understanding of health inequalities. We applied IPTW to report effect estimates on both absolute and relative scales to assess the strength of association. We also included 4 oral conditions, caries, periodontal disease, nonfunctional dentition, and edentulism within multimorbidity estimates, allowing dentate and edentate populations to be analyzed. Furthermore, clinical oral health data were used, increasing the reliability of results. However, study weaknesses are also acknowledged and provide a further point of future research. Our inequality estimates may be underestimated due to the more advantaged study population. There was underrepresentation of people experiencing social disadvantage in complete case analysis; therefore, our findings on social inequalities in multimorbidity are biased toward null, or conservative. Demographic and medical data were self-reported, and therefore misclassification is possible.

In response to the growing burden of multimorbidity, it has become necessary to reevaluate existing primary care pathways, which tend to promote a singular, specialist health model (Academy of Medical Sciences 2018). Consequently, as we move forward to a more integrated health system and adopt a more person-centered approach, we hope that oral health is also prioritized within health policy. From a clinical perspective, the delivery of key oral health messages and preventive oral advice within health care settings provides an important opportunity to support the well-being of multimorbid populations, through the provision of good oral health. Tackling health inequalities also remains an important policy directive, to actively reduce the burden of social disadvantage. Applicable to both general and oral health, it is important that policy makers address the social determinants of health and the subsequent common risk factors, to drive positive health change among those who experience social disadvantage (WHO 2023).

## Conclusion

This study has taken a novel approach to health inequalities by including oral conditions in the general compendium of health disorders. Whilst the limitations of this study are well recognized, we hope this study provides an important starting point for further multimorbidity research, inclusive of chronic oral diseases. By conducting this study, we have challenged existing views on multimorbidity and brought chronic oral conditions to the forefront of the multimorbidity agenda.

## Author Contributions

A. Mirza, contributed to conception, design, data acquisition, analysis, and interpretation, drafted and critically revised the manuscript, R.G. Watt, A. Heilmann, M. Stennett, A. Singh, contributed to conception, design, data analysis and interpretation, critically revised the manuscript. All authors gave final approval and agree to be accountable for all aspects of the work.

## Supplemental Material

sj-docx-1-jdr-10.1177_00220345241228834 – Supplemental material for Social Disadvantage and Multimorbidity Including Oral Conditions in the United States Supplemental material, sj-docx-1-jdr-10.1177_00220345241228834 for Social Disadvantage and Multimorbidity Including Oral Conditions in the United States by A. Mirza, R.G. Watt, A. Heilmann, M. Stennett and A. Singh in Journal of Dental Research
