# Supplementary material for: Social Disadvantage and Multimorbidity Including Oral Conditions in the United States
Source: J Dent Res. 2024 Mar 19;103(5):477–83. doi: 10.1177/00220345241228834 (PMC11047010; doi:10.1177/00220345241228834)
Supplement: sj-docx-1-jdr-10.1177_00220345241228834 – Supplemental material for Social Disadvantage and Multimorbidity Including Oral Conditions in the United States [file sj-docx-1-jdr-10.1177_00220345241228834.docx]

Appendix:

Social Disadvantage and Multimorbidity Including Oral Conditions in the USA

Afshan Mirza^1^, Richard G Watt^1^, Anja Heilmann^1^, Michelle Stennett^1^, and Ankur Singh^2,3^**.**

^1^Department of Epidemiology and Public Health, University College London, London, United Kingdom.

^2^Centre for Epidemiology and Biostatistics, Melbourne School of Population and Global Health, University of Melbourne, Melbourne, Victoria, Australia.

^3^Melbourne Dental School, University of Melbourne, Melbourne, Victoria, Australia.

| **STROBE CHECKLIST** | Item No | Recommendation |
| --- | --- | --- |
| **Title and abstract** | 1 | (*a*) Indicate the study’s design with a commonly used term in the title or the abstract:  Study design documented in abstract |
|  |  | (*b*) Provide in the abstract an informative and balanced summary of what was done and what was found:  300 word abstract included |
| Introduction | | |
| Background/rationale | 2 | Explain the scientific background and rationale for the investigation being reported:  Introduction section |
| Objectives | 3 | State specific objectives, including any prespecified hypotheses:  Introduction section |
| Methods | | |
| Study design | 4 | Present key elements of study design early in the paper:  Materials and methods: Data and analytical sample section |
| Setting | 5 | Describe the setting, locations, and relevant dates, including periods of recruitment, exposure, follow-up, and data collection:  Materials and methods: Data and analytical sample section  Participant flow diagram: Appendix Figure 2 |
| Participants | 6 | (*a*) Give the eligibility criteria, and the sources and methods of selection of participants:  Materials and methods: Data and analytical sample section  Participant flow diagram: Appendix Figure 2 |
| Variables | 7 | Clearly define all outcomes, exposures, predictors, potential confounders, and effect modifiers. Give diagnostic criteria, if applicable:  Materials and methods: Outcomes; Exposure; Covariates section  Appendix Figure 3 |
| Data sources/ measurement | 8* | For each variable of interest, give sources of data and details of methods of assessment (measurement). Describe comparability of assessment methods if there is more than one group:  Material and methods: Outcome; Exposure; Covariates section  Appendix Figure 3 |
| Bias | 9 | Describe any efforts to address potential sources of bias:  Oral health data collected by calibrated dental professionals  E-Values provided for relative risk estimates |
| Study size | 10 | Explain how the study size was arrived at:  Appendix Figure 2 |
| Quantitative variables |  | Explain how quantitative variables were handled in the analyses. If applicable, describe which groupings were chosen and why:  Materials and methods: Outcome; Exposure; Covariates section |
| Statistical methods | 12 | (*a*) Describe all statistical methods, including those used to control for confounding:  Materials and methods: Statistical analyses section |
|  |  | (*b*) Describe any methods used to examine subgroups and interactions:  Analysis of two multimorbid populations: (a) those with medical conditions, (b) those with medical and dental conditions |
|  |  | (*c*) Explain how missing data were addressed:  Materials and methods: Statistical analyses section |
|  |  | (*d*) If applicable, describe analytical methods taking account of sampling strategy:  Materials and methods: Statistical analyses section |
|  |  | (*e*) Describe any sensitivity analyses:  Comparison of characteristics for those included and excluded in the population sample  E-Values provided for relative risk estimates ( |
| Results | | |
| Participants | 13* | (a) Report numbers of individuals at each stage of study—e.g. numbers potentially eligible, examined for eligibility, confirmed eligible, included in the study, completing follow-up, and analysed:  Appendix Figure 2: Participant flow diagram  Results section |
|  |  | (b) Give reasons for non-participation at each stage:  Materials and methods: Data and analytical sample section  Appendix Figure 2: Participant flow diagram |
|  |  | (c) Consider use of a flow diagram:  Appendix Figure 2: Participant flow diagram |
| Descriptive data | 14* | (a) Give characteristics of study participants (e.g. demographic, clinical, social) and information on exposures and potential confounders:  Results section  Table 1 (results section) |
|  |  | (b) Indicate number of participants with missing data for each variable of interest:  Appendix Figure 2: Participant flow diagram |
| Outcome data | 15* | Report numbers of outcome events or summary measures:  Results section |
| Main results | 16 | (*a*) Give unadjusted estimates and, if applicable, confounder-adjusted estimates and their precision (e.g. 95% confidence interval). Make clear which confounders were adjusted for and why they were included:  Results section  Table 2 (results section) |
|  |  | (*b*) Report category boundaries when continuous variables were categorized:  n/a |
|  |  | (*c*) If relevant, consider translating estimates of relative risk into absolute risk for a meaningful time period:  Results section – Absolute and relative risk estimates provided  Table 2 (results section) |
| Other analyses | 17 | Report other analyses done—e.g. analyses of subgroups and interactions, and sensitivity analyses:  Bias analysis  Sensitivity analysis |
| Discussion | | |
| Key results | 18 | Summarise key results with reference to study objectives:  Discussion section covers points 18-21 |
| Limitations | 19 | Discuss limitations of the study, taking into account sources of potential bias or imprecision. Discuss both direction and magnitude of any potential bias |
| Interpretation | 20 | Give a cautious overall interpretation of results considering objectives, limitations, multiplicity of analyses, results from similar studies, and other relevant evidence |
| Generalisability | 21 | Discuss the generalisability (external validity) of the study results |
| Other information | | |
| Funding | 22 | Give the source of funding and the role of the funders for the present study and, if applicable, for the original study on which the present article is based:  Funding statement included |

*Give information separately for exposed and unexposed groups.

**Appendix Figure 1: STROBE checklist**

Total number of participants invited to NHANES (2013-2014)

**n=14 332**

Included study participants

**n=10 175**

Participants aged 0-29 years (n=5362) excluded from analysis as no periodontal data

**n=4813**

Percentage of missing data on variables of interest

Education 0.1%

Income 6.6%

Smoking 3.7%

Multimorbidity 7.9%

Multimorbidity including oral conditions 10.7%

Observations with missing data on at least one variable of interest excluded (n=1120)

**Final sample size n=3693**

**Appendix Figure 2: Participant flow diagram for NHANES (2013-2014)**


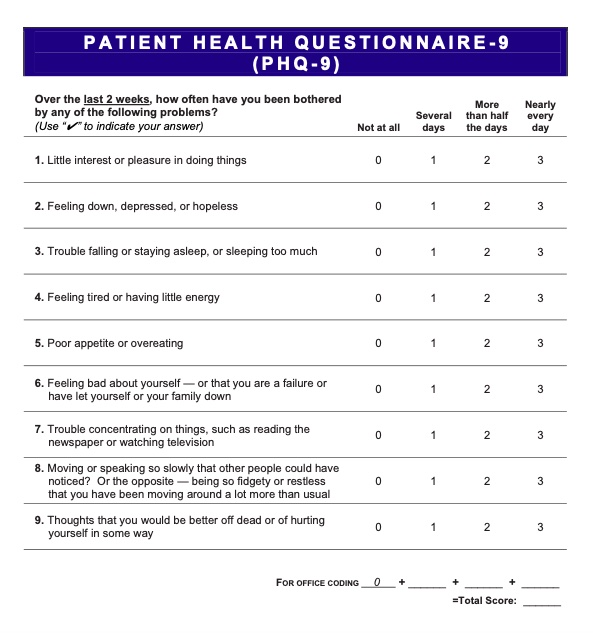


**Appendix Figure 3: Patient Health Questionnaire-9 (PHQ-9) for Depression**

**Appendix Table 1:** Comparison of sample characteristics between observations with no missing data and observations with missing data on at least one variable of interest (NHANES 2013-2014; weighted %)

|  | Observations with missing data | Complete case analysis |
| --- | --- | --- |
|  | *n*=1120 (%) | *n*=3693 (%) |
| Multimorbidity  Excluding chronic oral conditions  Including chronic oral conditions  Income* | 19.8  51.5 | 20.8  53.4 |
| High | 21.8 | 28.9 |
| Medium | 58.0 | 58.2 |
| Low income | 20.2 | 12.9 |
|  |  |  |
| Education* |  |  |
| High | 63.1 | 65.3 |
| Medium | 21.5 | 21.1 |
| Low | 15.4 | 13.6 |
|  |  |  |
| Sex* |  |  |
| Male | 41.0 | 48.9 |
| Female | 59.0 | 51.1 |
|  |  |  |
| Age |  |  |
| 30-39 | 22.3 | 21.3 |
| 40-49 | 25.1 | 22.2 |
| 50-64 | 30.3 | 33.3 |
| 65+ | 22.3 | 23.2 |
|  |  |  |
| Ethnicity* |  |  |
| White | 54.9 | 55.1 |
| Mexican | 19.9 | 16.1 |
| Black | 13.8 | 16.5 |
| Other | 11.4 | 12.3 |
|  |  |  |
| Marital status |  |  |
| Single | 36.6 | 32.0 |
| Married | 63.4 | 68.0 |
|  |  |  |
| Smoking status* |  |  |
| No | 44.2 | 54.1 |
| Yes | 27.2 | 19.3 |
| Ex-smoker | 28.6 | 26.6 |

*p value <0.05
